# Knowledge, attitude and compliance towards travel vaccines among Nigerian travellers at an international airport

**DOI:** 10.4102/phcfm.v11i1.2063

**Published:** 2019-11-11

**Authors:** Babatunde A. Akodu, Fanny O. Ogwu, Abdul-Hakeem O. Abiola

**Affiliations:** 1 Department of Community Health and Primary Care, College of Medicine, University of Lagos, Lagos, Nigeria; 2 Port Health, Federal Airports Authority of Nigeria, Lagos, Nigeria

**Keywords:** travel-related diseases, travel vaccines, travellers attitudes, infectious diseases

## Abstract

**Background:**

The risk of contracting a travel-related disease does not only depend on the destination of travel and length of the trip, but also on the traveller’s own health status. Travel vaccines avert the increase of communicable disease. Awareness of traveller’s behaviours and their attitudes concerning infectious diseases can inform policy aimed at protecting the individual travellers, their contacts and the communities into which they travel.

**Aim:**

This study on travel vaccine was aimed to determine the level of knowledge, attitude and compliance to travel vaccines.

**Setting:**

This study was conducted among Nigerian travellers at Murtala Mohammed International Airport, Ikeja, Nigeria.

**Methods:**

It was a cross-sectional descriptive study using systematic sampling technique, with the aid of interviewer-administered questionnaire to select 198 respondents for the study. The statistical software EPI-Info 7 was used for data analysis.

**Results:**

The mean age of respondents was 35.34 + 9.91 years and majority (58.1%) of the respondents were men. Majority (54.0%) were married and (43.4%) had tertiary education. About 35.9% were travelling to other African countries, 9.6% to Middle Eastern countries, 16.2% to Europe, 13.6% to North America and 7.6% to Australia. Their main purpose of travel was for employment or working (22.2%), business activities (20.7%), touring (16.2%) and visiting families and relatives (15.7%). About a half (41.4%) had good knowledge of travel vaccines, majority (83.8%) had positive attitude and majority (66.2%) had been vaccinated for yellow fever before travel.

**Conclusion:**

Significant associations exist between tribe, religion, education and knowledge of travel vaccines.

## Introduction

Over the last few decades, international travel has increased significantly in magnitude, speed and geographical reach, with 940 million arrivals reported worldwide in 2010.^1^ It exposes travellers to new culture, physiological and microbiological experiences.^2^ With the world in motion, a disease is a trip away.^2^

Travellers cause the spread of infectious diseases across international borders through the frequency of their travel and exposure to insect bites.^3^ The danger of exposure to a travel-related disease is not only determined by the destination of travel, length of the trip and planned activities, but also by the traveller’s own health status.^4^ Travel may be the single determinant for the spread of communicable diseases that are well handled in the traveller’s country of residence, especially vaccine-preventable infections such as hepatitis A, typhoid fever, poliomyelitis, yellow fever and measles.^4^ The awareness of immunisation among travellers is a vital measure for the control of travel-associated infectious diseases.

Awareness of travellers’ behaviours and their attitudes towards communicable diseases can inform policy aimed at protecting the individual travellers, their contacts and the communities into which they travel. The awareness of control measures on minimal health risk during travel is low among Nigerian travellers.^2^

Travellers’ abilities to adjust, handle and survive are affected by factors like personality and experience, which vary according to age, sex, tradition, social, education and well-being.^5^ Relatively little is identified concerning travellers’ knowledge and perception of health risks connected with travel and how to use protective measures prior to and while travelling abroad.

Although the bulk of travel-associated infectious diseases can be prevented, the public health load of these diseases remains considerable. Travellers are at growing threat of contact to travel-related health challenges, as well as infectious diseases, which may be imported back to their country of residence.^6^

A research conducted in Japan, from April 2007 to May 2008, on knowledge, attitude and practices of Japanese travellers on disease threats and immunisation uptake revealed that majority (87.4%) of the participants sought general information on their destination, few (38.7%) sought for travel health information and very few (2%) received the information from travel medicine specialists.^7^ More than half were either ignorant of the risks or believed that there was no threat of typhoid fever, hepatitis A and hepatitis B in their destination. Half of the participants stated that vaccines enable adequate guard and a minority of participants (13.6%) stated that vaccines were safe. Fewer than 10% had received the vaccines for the vaccine-preventable diseases. Therefore, specialised travel health services was needed in Japan.^7^

Circumstances whereby travellers are denied access at the port of entry because of possession of fake yellow cards as experienced by 125 Nigerians including a serving senator in March 2012 from South Africa were embarrassing.^8^

Descriptive study carried out in Nigeria in 2013 among 189 medical doctors of the University of Jos Teaching Hospital showed that the majority of participants (96.3%) were aware of travel vaccines, with 45 of the 63 participants (71.3%) who had proceeded on international travel prior to the research had received travel vaccines in their last travel. Knowledge of travel vaccine was found to have statistically significant association with uptake of travel vaccine.^6^

This research showed the justification to address the knowledge and uptake of travel vaccine among physicians. When a traveller is infected in anyway, it may interfere with his or her planned activities. Alteration in planned activities may occur following an infection in a traveller. This may give rise to frustration.

A research carried out on departing travellers’ knowledge of, attitudes towards and practices on the prevention of infectious diseases at the Johannesburg International Airport revealed the mean age of travellers as 42 years, with one-third being aged 50 years or above. The commonest travel reasons indicated by participants were leisure (42%) and business (37%); few (8%) of the subjects stated they were visiting their friends or relatives. About three-quarter of the participants were able to produce a valid vaccination certificate to yellow fever-endemic countries. About one-fifth (22%) of travellers to countries not endemic for yellow fever had been specifically immunised against yellow fever for their journeys.^9^

The objective of this research is to evaluate the knowledge of, attitude towards and compliance to travel vaccines among Nigerian travellers to prevent importation and exportation of communicable disease.

The significance of this research is the critical need on how to decrease the exportation and importation of infectious diseases for a better humanity. A pre-travel consultation with health professionals can provide the international traveller with the essential protective information on minimising health threats during travel, as well as the threats of infectious diseases and chance for appropriate vaccination and chemoprophylaxis. These vaccines are administered for protection from new infections in the region to be visited and also to prevent the transportation of diseases from one place to another. Travellers who search for pre-travel health information from health specialists possess good knowledge of infectious disease treatments, more correct perceptions and an elevated stage of intended risk-reducing behaviors.^3^ Administration of travel vaccines is valuable to public health and the community because it guides against the increase of diseases, guarantees life and improves human health through planned efforts and informed choices of society and organisations.

There is a paucity of data regarding the knowledge of, attitude towards and compliance to travel vaccines among travellers at the port of travel. Risk reduction through sensitisation and preventive measures, such as updating of immunisation record on the vaccination travel certificate especially before embarking on international trips, may improve the knowledge and compliance of travellers.

In view of the above, the introduction of yellow card was made. This is an International Certificate of Vaccination (ICV) produced by the World Health Organization.^8^ This is accepted internationally and may be essential for access to some countries with high health threats for travellers.

We believe that the findings of this study might help highlight reasons for enforcing travel vaccines among travellers, and to quarantine and treat travellers confirmed to be carrier to prevent the spreading of diseases.

## Methods

The Murtala Mohammed International Airport, Ikeja, is Nigeria’s premier international air gateway.^10^ The current international airport terminal was built and commissioned over 40 years ago, in 1978. The terminal opened officially on 15 March 1979. Formerly known as Lagos International Airport, it was, however, renamed after the late Nigerian Head of State, General Murtala Muhammed, who died in 1976.^10^

There are around 40 airlines that fly from Lagos to over 60 destinations worldwide, carrying over 10 million passenger every year. The Lagos Nigerian Airport Code is LOS and it is the busiest airport in Nigeria.

An average of 1500 travellers travel daily at the Murtala Mohammed International Airport, of which 1100 travel to countries where International Health Certificate is required before entry.

A cross-sectional descriptive study was conducted among 206 international travellers prior to their departure from Murtala Mohammed International Airport in Ikeja, Lagos, Nigeria.

Inclusion criteria for the study were as follows:

- Travellers that are 18 years and above.
- All gender, that is, males and females.
- Nigerian passengers boarding flights to countries that require valid travel vaccines; Nigerians travelling to other African countries, Asia and Australia and Middle Eastern countries at the point of departure from the international airport in Ikeja.

Exclusion criteria for the study were as follows:

- arriving travellers at the airport
- non-Nigerian travellers.

### Sampling technique

From the average number of 1100 travellers visiting countries where International Health Certificate is required before entry, 206 travellers were sampled for this study through systematic sampling technique. The sample frame was 1100/206 = 5.3 (sampling interval).

As the travellers arrived at the departure hall from the check-in counter, the first traveller was selected randomly, and then every 5th traveller was selected for this study.

### Instruments for the study

The instrument that was utilised for this study was interviewer-administered questionnaire which was adapted from the review of past studies. The questionnaire was divided into four sections. Section A was on socio-demographic characteristics, Section B was on the knowledge of travel vaccines, Section C was on the attitude towards travel vaccines and Section D was on compliance towards travel vaccines.

### Data collection procedure

The use of interviewer-administered questionnaire was used in the collection of data for this study, which was collected at the point of departure. A 2-week timeframe was used in the collection of data with the help of two research assistants.

### Data analysis

#### Scoring system

All relevant knowledge questions were scored to assess the level of knowledge of international health certificate. Each correct option was assigned 1 mark. The total scores were converted into percentage. The cut-off score used was 50%. Scores between 0% and 49% were considered poor, while scores between 50% and 100% were considered good. For the attitude, scores between 0 and 49 were considered negative, while scores between 50 and 100 were considered positive.

The statistical software EPI-Info 7 was used for data analysis. Descriptive statistics were calculated for all variables. For continuous variables, mean and standard deviation were calculated. Frequency distribution and cross-tabulations were generated using chi-squared test to compare proportions, where cells were less than 5, Fisher’s exact test was calculated and *p*-value was set at 0.05.

### Ethical consideration

Ethical approval for the study was obtained from LUTH Ethic Committee; permission was obtained from the management of Murtala Muhammed International Airport, Ikeja, Lagos, stating the purpose of the study.

## Results

From the data in Table 1, 35.9% of the respondents indicated that they were travelling to other African countries, few (9.6%) to Middle Eastern countries, 16.2% to Europe, 13.6% to North America and 7.6% to Australia.

**TABLE 1:** Travel characteristics of respondents.

Variable	Frequency (*n* = 198)	%
**Respondents’ travel destinations**		
Africa	71	35.9
Middle East	19	9.6
Europe	32	16.2
South America	23	11.6
North America	27	13.6
Australia	15	7.6
Asia	5	2.5
Others	6	3.0
**Frequency of air travel by respondents**		
Once	36	18.2
Twice	69	34.8
Thrice	20	10.1
More than thrice	73	36.9
**Purpose of travel**		
Employment or working	44	22.2
Studying	33	16.7
Attending business activity	41	20.7
Attending conference	17	8.6
Touring	32	16.2
Visiting families and relatives	31	15.7

Of the travellers, 18.2% were first-time air travellers, 34.8% indicated to have travelled by air twice and 36.9% had travelled by air for more than three times. Their main purpose of travel was for employment or working (22.2%), attending business activities (20.7%), touring (16.2%) and visiting families and relatives (15.7%).

From the data in Table 2, majority (83.8%) of the respondents indicated to have heard of yellow fever vaccine, while 16.2% were not aware; the major sources of information about yellow fever vaccine were through family and friends (35.5%), TV/radio/newspaper (17.5%) and hospital (19.9%). The majority (69.7%) of respondents were not aware of the causes of yellow fever. Less than a quarter (24.1%) of the respondents indicated that yellow fever vaccine will start providing protection immediately after receiving it, 24.7% indicated between 1 and 2 days and 28.9% stated that it will start protection after 10 days. Some (29.5%) of the respondents indicated that yellow fever vaccine will provide protection for between 4 and 5 years, 38.6% of the respondents indicated between 6 and 10 years and 19.9% of the respondents indicated more than 10 years. Regarding the knowledge of the diseases that can be prevented by vaccination, almost all (92.2%) respondents indicated yellow fever, 96.4% indicated polio, 59.6% indicated cholera and 57.8% indicated meningitis.

**TABLE 2:** Knowledge of health certificate and travel vaccines.

Variable	Frequency (*n* = 198)	%
**Heard of yellow fever vaccine**		
Heard	166	83.8
Not heard	32	16.2
**First source of information about yellow fever (*n* = 166)**		
TV/radio/newspaper	29	17.5
Family and friends	59	35.5
Hospital	33	19.9
School	19	11.4
**Know causes of yellow fever**	26	15.7
Yes	60	30.3
No	138	69.7
**Know when the yellow fever vaccine will start providing protection (*n* = 166)**		
Immediately after receiving it	40	24.1
Some hours after	19	11.4
Between 1 and 2 days	41	24.7
Between 3 and 5 days	5	3.0
Between 6 and 9 days	13	7.8
From 10 days onwards	48	28.9
**Know number of years the yellow fever vaccine will provide protection up to (*n* = 166)**		
Less than 1 year	7	4.2
Between 2 and 3 years	13	7.8
Between 4 and 5 years	49	29.5
Between 6 and 10 years	64	38.6
More than 10 years	33	19.9
**Diseases can be prevented by vaccination (*n* = 166)**†		
Hepatitis A	39	23.5
Hepatitis B	58	34.9
Yellow fever	153	92.2
Typhoid fever	84	50.6
Cholera	99	59.6
Polio	160	96.4
Rabies	87	52.4
Meningitis	96	57.8
Influenza	33	19.9
**Overall knowledge of travel vaccines**		
Good	82	41.4
Poor	116	58.6

† , Multiple responses.

On the overall scores of knowledge of travel vaccines, less than half (41.4%) of the respondents had good knowledge, while the majority (58.6%) had poor knowledge regarding diseases that can be prevented by vaccination.

Table 3 shows the attitude of respondents towards health certificate and yellow fever vaccines. From the data in the table, majority (52.0%) of the respondents were of the view that yellow fever vaccine is effective in providing protection, 56.1% indicated that yellow fever vaccine is safe, 53.0% agreed that the certificate of yellow fever vaccination should be received before travel and almost half of the respondents (49.5%) agreed with the view that no traveller should be allowed to board without travel vaccine certificate.

**TABLE 3:** Attitude towards health certificate and travel vaccines.

Variable	Strongly agree		Agree		Neutral		Disagree		Strongly disagree	
	Freq	%	Freq	%	Freq	%	Freq	%	Freq	%
Yellow fever vaccine is effective in providing protection	60	30.3	103	52.0	34	17.2	1	0.5	-	-
Yellow fever vaccine is safe	49	24.7	111	56.1	38	19.2	-	-	-	-
The certificate of yellow fever vaccination should be received before travel	51	25.8	105	53.0	35	17.7	6	3.0	1	0.5
No traveller should be allowed to board without travel vaccine certificate.	30	15.2	98	49.5	47	23.7	20	10.1	3	1.5
If I travel frequently, I do not need preventive measures much	25	12.6	52	26.3	58	29.3	52	26.3	11	5.6
It is important to visit a doctor before travel	34	17.2	90	45.5	48	24.2	22	11.1	4	2.0
Looked for health-related information about travel areas	31	15.7	101	51.0	58	29.3	6	3.0	2	1.0
I think that travel does not increase risk if I am in good health	25	12.6	90	45.5	55	27.8	21	10.6	7	3.5
In my opinion, visiting a doctor before travel is essential	26	13.1	91	46.0	58	29.3	23	11.6	-	-
I think that vaccines are very important before travel	33	16.7	95	48.0	53	26.8	15	7.6	2	1.0
I think that malaria chemoprophylaxis is not effective	19	9.6	56	28.3	74	37.4	42	21.2	7	3.5
In my opinion, personal hygiene protects from many diseases	45	22.7	101	51.0	38	19.2	10	5.1	4	2.0
I do not think that infectious diseases could be related to travelling	17	8.6	64	32.3	55	27.8	46	23.2	16	8.1
Acquired information about potential travel hazards	32	16.2	97	49.0	62	31.3	6	3.0	1	0.5
Overall attitude towards travel vaccines										
Positive	166	83.8	-	-	-	-	-	-	-	-
Negative	32	16.2	-	-	-	-	-	-	-	-

Freq, frequency.

Some (26.3%) of the respondents agreed and disagreed, respectively, that frequent travellers do not need preventive measures much, 45.5% agreed that it is important to visit a doctor before travel, 51.0% agreed to look for health-related information about travel areas, 48.0% stated that vaccines are very important before travel and 49.0% agreed with the view of acquiring information about potential travel hazards.

On the overall attitude towards travel vaccines, the majority (83.8%) of respondents had positive attitude, while few (16.2%) had negative attitude. Right (appropriate) attitudes are positive, while wrong (inappropriate) attitudes are negative.

From the data in Table 4, 66.2% of the respondents indicated to have been vaccinated for yellow fever, while 33.8% had not been vaccinated. On the time of vaccination, 64.1% had been vaccinated for over 6 months ago, while 24.4% had been vaccinated for less than 10 days before travel. Also, 87.0% of those who were vaccinated indicated that they got certificate for vaccination after vaccination, while 13.0% indicated that they did not receive such certificates.

**TABLE 4:** Compliance towards international health certificate and travel vaccines.

Variable	Frequency (*n* = 198)	%
**Vaccination for yellow fever**		
Vaccinated	131	66.2
Not vaccinated	67	33.8
**Time vaccinated before travel (*n* = 131)**		
Less than 10 days	32	24.4
Less than 6 months	15	11.5
Over 6 months ago	84	64.1
**Got certificate for vaccination after being vaccinated (*n* = 131)**		
Yes	114	87.0
No	17	13.0
**Frequency of going for vaccination (*n* = 131)**		
Anytime I want to travel out of the country	24	18.3
When I want to travel to countries where vaccination is required	86	65.6
Before and after travelling out of the country	21	16.1
**Person who recommended yellow fever vaccination (*n* = 131)**		
Doctor	26	19.8
Friends	21	16.0
At the embassy	68	51.9
Others	16	12.3
**Reason for vaccination of yellow fever (*n* = 131)**		
For prevention against yellow fever	34	26.0
To meet the mandatory requirement of vaccination as imposed by the country of destination	64	48.9
Advised by the physician	8	6.1
Advised by the embassy	25	19.0
**Diseases vaccinated for (*n* = 131)**		
Hepatitis A	18	13.7
Hepatitis B	17	13.0
Yellow fever	120	91.6
Typhoid fever	28	21.4
Cholera	33	25.2
Polio	110	84.0
Rabies	19	14.5
Meningitis	23	17.6
Influenza	9	6.9
**If not vaccinated, did you get vaccination certificate (*n* = 67)**		
Yes	4	6.0
No	63	94.0

The majority (65.6%) of respondents indicated that they got vaccination whenever they travel to countries that require vaccination, while 18.3% got vaccination anytime they travel out of the country. The majority (51.9%) of respondents indicated that yellow fever vaccination was recommended at the embassy, 19.8% mentioned about doctor and 16.0% indicated friends. The vast majority (91.6%) of respondents indicated to have been vaccinated for yellow fever, 84.0% indicated polio, 25.2% indicated cholera and 17.7% indicated meningitis. Of those who did not have vaccination, few (6.0%) indicated that they had vaccination certificate.

From the data in Table 5, it is shown that there was a statistically significant association between some variables of socio-demographic characteristics (tribe, religion and education) and the knowledge of travel vaccines (*p* < 0.05). Oldest respondents (64.7%), male gender (45.2%), Yoruba respondents (49.5%), married respondents (45.8%), Christianity (45.7%) and tertiary education (55.8%) were more knowledgeable about travel vaccines than other respondents.

**TABLE 5:** Association between knowledge of travel vaccines and socio-demography of respondents.

Variable	Knowledge of travel vaccines				*χ*^2^	*p*
	Poor (*n* = 116)		Good (*n* = 82)			
	*n*	%	*n*	%		
**Age (years)**						
≤ 20	7	53.8	6	46.2	0.602	0.963
21–30	36	62.1	22	37.9	-	-
31–40	42	58.3	30	41.7	-	-
41–50	23	57.5	17	42.5	-	-
51–60	8	53.3	7	46.7	-	-
**Gender**						
Male	63	54.8	52	45.2	1.636	0.201
Female	53	63.9	30	36.1	-	-
**Tribe**						
Yoruba	55	50.5	54	49.5	8.780	0.028*
Igbo	47	66.2	24	33.8	-	-
Hausa	5	62.5	3	37.5	-	-
Others	9	90.0	1	10.0	-	-
**Marital status**						
Single	46	59.7	31	40.3	-	0.279*
Married	58	54.2	49	45.8	-	-
Separated	6	85.7	1	14.3	-	-
Divorced	3	100.0	0	0	-	-
**Widowed/widower**	3	75.0	1	25.0	-	-
**Religion**						
Christianity	82	54.3	69	45.7	-	0.048*
Islam	33	71.7	13	28.3	-	-
Traditional	1	100.0	0	0	-	-
**Education**						
Primary	6	75.0	2	25.0	19.451	0.001
Secondary	30	85.7	5	14.3	-	-
Post-secondary	34	63.0	20	37.0	-	-
Tertiary	38	44.2	48	55.8	-	-
Others	8	53.3	7	46.7	-	-

s.d., standard deviation.

Poor, Mean ± s.d. 35.28 + 9.85; Good, 35.43 + 10.04; *t* = 0.105; *p*, 0.916.

* , Fisher’s exact test.

Table 6 shows that there was no statistically significant association between socio-demographic characteristics and attitude towards travel vaccines (*p* > 0.05) except for marital status (*p* < 0.05). Oldest respondents (80.0%), female gender, other tribe (90.0%) and other educational qualifications had more positive attitude towards travel vaccines than other respondents.

**TABLE 6:** Association between attitude towards travel vaccines and socio-demography of respondents.

Variable	Attitude towards travel vaccine				*χ*^2^	*p*
	Poor (*n* = 32)		Good (*n* = 166)			
	*n*	%	*n*	%		
**Age (years)**						
≤ 20	2	15.4	11	84.6	-	0.476*
21–30	7	12.1	51	87.9	-	-
31–40	10	13.9	62	86.1	-	-
41–50	10	25.0	30	75.0	-	-
51–60	3	20.0	12	80.0	-	-
**Gender**						
Male	21	18.3	94	81.7	-	0.435*
Female	11	13.3	72	86.7	-	-
**Tribe**						
Yoruba	14	12.8	95	87.2	-	0.333*
Igbo	15	21.1	56	78.9	-	-
Hausa	2	25.0	6	75.0	-	-
Others	1	10.0	9	90.0	-	-
**Marital status**						
Single	8	10.4	69	89.6	-	0.043*
Married	19	17.8	88	82.2	-	-
Separated	3	42.9	4	57.1	-	-
Divorced	0	0	3	100.0	-	-
**Widowed/widower**	2	50.0	2	50.0	-	-
**Religion**						
Christianity	25	16.6	126	83.4	-	1.000*
Islam	7	15.2	39	84.8	-	-
Traditional	0	0	1	100.0	-	-
**Education**						
Primary	1	12.5	7	87.5	-	0.089*
Secondary	10	28.6	25	71.4	-	-
Post-secondary	10	18.5	44	81.5	-	-
Tertiary	11	12.8	75	87.2	-	-
Others	0	0	15	100.0	-	-

s.d., standard deviation.

Poor, Mean ± s.d. 37.47 ± 10.54; Good, 34.93 ± 9.76; *t* = 1.331; *p*, 0.185.

* , Fisher’s exact test.

Table 7 shows that there was no statistically significant association between socio-demographic characteristics and yellow fever vaccination among respondents (*p* > 0.05) except for gender and marital status (*p* < 0.05).

**TABLE 7:** Association between vaccination for yellow fever vaccines and socio-demography of respondents.

Variable	Vaccination for yellow fever				*χ*^2^	*p*
	Vaccinated (*n* = 131)		Not vaccinated (*n* = 67)			
	*n*	%	*n*	%		
**Age (years)**						
≤ 20	10	76.9	3	23.1	2.408	0.672
21–30	34	58.6	24	41.4	-	-
31–40	49	68.1	23	31.9	-	-
41–50	28	70.0	12	30.0	-	-
51–60	10	66.7	5	33.3	-	-
**Gender**						
Male	83	72.2	32	27.8	4.429	0.035
Female	48	57.8	35	42.2		
**Tribe**						
Yoruba	71	65.1	38	34.9	-	0.194*
Igbo	46	64.8	25	35.2	-	-
Hausa	8	100.0	0	0	-	-
Others	6	60.0	4	40.0	-	-
**Marital status**						
Single	49	63.6	28	36.4	-	0.040*
Married	72	67.3	35	32.7	-	-
Separated	7	100.0	0	0	-	-
Divorced	0	0.0	3	100.0	-	-
**Widowed/widower**	3	75.0	1	25.0	-	-
**Religion**						
Christianity	102	67.5	49	32.5	-	0.654*
Islam	28	60.9	18	39.1	-	-
Traditional	1	100.0	0	0	-	-
**Education**						
Primary	4	50.0	4	50.0		0.833*
Secondary	23	65.7	12	34.3	-	-
Post-secondary	37	68.5	17	31.5	-	-
Tertiary	58	67.4	28	32.6	-	-
Others	9	60.0	6	40.0	-	-

s.d., standard deviation.

Vaccinated, Mean ± s.d. 35.69 + 10.06; Not vaccinated, 34.66 ± 9.64; *t* = 0.691, *p*, 0.490.

* , Fisher’s exact test.

## Discussion

From the findings of this study, it was shown that the mean age of the respondents was 35.34 ± 9.91 years, 29.3% of the respondents were between 21 and 30 years of age, 36.4% were between 31 and 40 years of age, and 7.6% were between 51 and 60 years of age. Similarly, a study conducted in Nigeria among travellers showed a mean age of 33.8 ± 4.5 years.^11^ Another study conducted in Saudi Arabia showed that the mean age of the studied travellers was 33 ± 10.1 years.^12^ In contrast, a higher mean age (42 years) was recorded in a similar study conducted among travellers in South Africa and 30% of the travellers were 50 years and above.^9^ This showed that the South African travellers were older than the travellers of this study. The reason for the differences might be that many young Nigerians like to travel abroad with the hope of getting better opportunity to survive.

This study also showed that 58.1% of the respondents were men, while 41.9% were women. Similarly, a study conducted among travellers in Saudi Arabia reported that the majority (78.8%) were men.^12^ The similarity reported in this study can be attributed to the fact that more males tend to travel from their home to other places locally and internationally than their female counterparts. Also in this study, more than half (55.1%) of the travellers were Yoruba ethnic group, followed by Igbos (35.9%) and other tribes (5.1%). The reason for the majority recorded among the Yoruba tribe might be that the study location is in Lagos State, South Western Nigeria, where Yoruba tribe is dominant.

On the travel characteristics of respondents, it was shown from the results that 35.9% of the respondents were travelling to other African countries, few (9.6%) to Middle Eastern countries, 16.2% to Europe, 13.6% to North America and 7.6% to Australia. However, a similar study conducted in Dubai, UAE, revealed that the most frequently reported destinations were Asia (30.2%) and India (24.7%), followed by Africa (16%).^13^ Another study conducted among travellers in Singapore, Kuala Lumpur, Taipeh, Melbourne and Seoul revealed that their travel destinations were Asia (82%) and Western countries.^14^ The reason for the higher proportions of travellers to other African countries recorded from this study as against other studies could be attributed to the perception that journey by air is safer and faster than travel by road, and some travellers may have businesses in other African countries.

This study also showed that the respondents’ main purpose of travel was for employment or working (22.2%), attending business activities (20.7%), touring (16.2%) and visiting families and relatives (15.7%). Similarly, a study conducted among travellers at the Johannesburg International Airport showed that the reasons for travel among respondents included leisure (42%), business (37%) and visiting friends or relatives (8%).^9^

The variation in the purpose of travels between this study and the study conducted among Johannesburg International Airport travellers^15^ justified the earlier assumption that many Nigerian travellers, especially youth, travel out of the country for better future abroad, with some going for educational purposes because of preference for foreign education and exposure to modern technology.

Regarding the knowledge of health certificate and travel vaccines, the majority (83.8%) of respondents indicated to have heard of yellow fever vaccine – the major sources of information were through family and friends (35.5%). Overall, less than half (41.4%) of the respondents had good knowledge, while the majority (58.6%) had poor knowledge. Similarly, a study carried out to determine the level of travel health knowledge, attitudes and practices among Saudi travellers at international airports of KSA found poor knowledge of travel vaccines.^12^ Another study conducted to determine the level of the knowledge, attitudes and practices of Muscat International Airport travellers about travel health found a knowledge gap, the need for travel health services in Oman and inadequate level of travellers’ knowledge.^16^ In another study conducted among Chinese travellers who visit travel clinics in preparation for international travel, the knowledge about vaccine-preventable diseases was found to be low.^17^ However, in contrast to the findings of this study, a cross-sectional survey carried out in Jebel Ali Free zone’s Dubai, UAE, to assess the knowledge of, attitude towards and practice of travellers towards travel health found good knowledge regarding the importance of vaccination among these travellers (96.2%).^13^ The high proportions with poor knowledge of travel vaccine recorded in this study might be attributed to the fact that vaccination is not required in all countries, and some travellers might be going to countries where yellow fever vaccination is not required.

Regarding the attitude of respondents towards health certificate and travel vaccines, the majority (52.0%) respondents were of the view that yellow fever vaccine is effective in providing protection, 56.1% indicated that yellow fever vaccine is safe, 26.3% agreed and disagreed, respectively, that frequent travellers do not need preventive measures much. On the overall attitude towards travel vaccines, the majority (83.8%) of respondents had positive attitude, while few (16.2%) had negative attitude. Similarly, a study carried out among Saudi travellers at international airports of KSA reported that travellers had highly positive attitude regarding the importance of travel vaccine (84.1%), while others were not as high, for example, the importance of visiting a doctor before travel (50%) and the importance of vaccination (52.7%).^12^ Another study conducted among US travellers travelling to a high-risk malaria-endemic region found that travellers perceived malaria as a high health risk, with only 17% of travellers having positive attitude towards vaccines.^18^ In contrast to the findings of this study, a cross-sectional survey carried out in Jebel Ali Free zone’s Dubai, UAE, to assess the attitude of travellers towards travel health revealed that there was a low attitude (55.6%) towards vaccinations among respondents travellers.^13^ Another study conducted among Oman travellers reported that less than 25% of the travellers showed concern about travel vaccines, which translated to poor attitude towards travel vaccines.^16^ The high proportion of respondents with positive attitude from this study might be because they understood the importance of vaccination before travel, and the fear of being deported because of violation of laws related to travel vaccinations. This study revealed that 66.2% of respondents had been vaccinated for yellow fever, while 33.8% had not been vaccinated. On the time of vaccination, 64.1% had been vaccinated for over 6 months prior, and 24.4% had been vaccinated for less than 10 days before travel. Also, 87.0% of those who were vaccinated indicated that they got certificate for vaccination after vaccination, while 13.0% indicated that they did not receive certificate. Similarly, a study conducted among UAE travellers showed that 22.8% of respondents had sought pre-travel health advice from travel clinics and had received vaccines.^13^ Another study conducted among Oman travellers about travel health found poor utilisation of travel medicine services.^16^ Yet in another study that determined the travel health practices of US travellers, 36% of travellers sought travel health advice.^18^ This is lower to the proportion of those who were vaccinated in this study. A similar study on uptake of travel vaccine conducted in Plateau State, North Central Nigeria found that 71.4% of the 63 respondents who had embarked on international travel prior to the study had taken travel vaccine in their last travel.^11^ In another study conducted on vaccination practice among Chinese travellers, the overall acceptance rate of recommended vaccines was 68.7%, but yellow fever was accepted by 99.8% of the participants when recommended.^17^ In a study conducted among travellers at Saudi International Airport, only 19.9% of the participants consulted a doctor for their travel, 23.7% looked for health-related information and only 11.2% got such information, and 3.3% got vaccination.^12^

The high proportion of vaccinated travellers reported in this study might be attributed to the fact that they may have been informed before travel on the requirements, and the importance of vaccination before travel. Although another similar study^12^ recorded lower proportion of those who seek for pre-travel medical advice, this might be that the country is not seen as yellow fever endemic state.

## Limitations of study

The study location was Murtala Mohammed International Airport, Ikeja, Lagos. Airports are reserved environment and as such are not easily accessible to researchers and significantly most international flights are scheduled at night. This research only covered one of the six functional International Airports in Nigeria. The authors recommend that future research be conducted in other airports of the Nigeria.

### Recommendations

Based on the results of this study, the following recommendations are made:

1. The Federal Ministry of Health should design awareness campaigns on the importance and needs of travel vaccination among Nigerian travellers.
2. The Federal Ministry of Health should collaborate with all stakeholders in the travel industry by making it compulsory for all travellers to seek pre-travel medical consultation before embarking on foreign trips. This will limit the spread and infection of diseases acquired within and outside the country.
3. The Federal Airport Authority, the Ministry of Health and the security agencies in travel industry should always check for the authentication of yellow fever certification at the airports to eradicate the culture of having vaccination certification without vaccination.
4. Billboards, leaflets and newsprint should be made available in all Nigerian airports by the Nigeria Airport Authority to increase the level of awareness of yellow fever vaccination among current and intending international travellers.

### Further research

There is a strong need to investigate the factors that influence the knowledge and compliance of travellers towards travel vaccines. It should be expanded to include issues like attitudes of healthcare personnel – poor communication between providers and clients and cultural beliefs.

## Conclusion

The overall study findings were as follows: the mean age of the respondents was 35.34 ± 9.91 years, the majority of respondents were men, one-third were travelling to African countries, few (9.6%) to Middle Eastern countries, 16.2% to Europe, 13.6% to North America and 7.6% were travelling to Australia.

The majority of respondents stated to have heard of yellow fever vaccine, the major sources of information were identified as family and friends. The majority respondents had poor knowledge of travel vaccine. The majority of respondents had positive attitude towards travel vaccines, while few had negative attitude. The majority of respondents had been vaccinated for yellow fever. The majority of those who were vaccinated indicated that they had got certificate for vaccination.

There was an association between some variables of socio-demographic characteristics (tribe, religion and education) and knowledge of travel vaccines, but there was no statistically significant association between socio-demographic characteristics, variables and attitude towards travel vaccines; also, association did not exist between socio-demographic characteristics and yellow fever vaccination among respondents except for gender and marital status.
